# A Cross-Sectional Study on University Students’ Knowledge, Attitudes, and Practices Toward COVID-19 in the United Arab Emirates

**DOI:** 10.4269/ajtmh.20-0857

**Published:** 2020-11-23

**Authors:** Hayder Hasan, Veena Raigangar, Tareq Osaili, Noorieh E. Neinavaei, Amin N. Olaimat, Iman Aolymat

**Affiliations:** 1 Department of Clinical Nutrition and Dietetics, College of Health Sciences, University of Sharjah, Sharjah, United Arab Emirates;; 2 Research Institute of Medical and Health Sciences, University of Sharjah, Sharjah, United Arab Emirates;; 3 Department of Physiotherapy, College of Health Sciences, University of Sharjah, Sharjah, United Arab Emirates;; 4 Department of Nutrition and Food Technology, Faculty of Agriculture, Jordan University of Science and Technology, Irbid, Jordan;; 5 Department of Clinical Nutrition and Dietetics, Faculty of Applied Medical Sciences, The Hashemite University, Zarqa, Jordan;; 6 Department of Basic Medical Sciences, Faculty of Medicine, The Hashemite University, Zarqa, Jordan

## Abstract

The unprecedented coronavirus pandemic is hitting the whole world, including the United Arab Emirates. Public awareness and adherence to the recommendations play a major role in managing a crisis of this magnitude which is largely affected by knowledge, attitudes, and practices (KAP). Hence, the aim of this study was to assess COVID-19–related KAP of the University of Sharjah (UOS) students and compare between health-related (HR) and non-HR (NHR) majors. A cross-sectional study was conducted in May 2020 in which 1,012 (481 health-related and 531 NHR) students participated via an online KAP questionnaire. The students’ sociodemographic characteristics and sources of information were also recorded, and data were analyzed. Students were aged 20–25 years, with an overall knowledge score of 72.4%, and the main source of their information was the Internet and social media (85.2%). Those in HR majors had a higher knowledge score (76%) than those in NHR students (69%). Regarding attitudes, both HR and NHR students demonstrated comparable and positive attitudes to curb the spread. With respect to practices, more NHR students used masks (92.3%), almost all the time than HR students (88.4%). HR students (99.4%) avoided crowded places and practiced social distancing more than NHR students (99.4% versus 97.4% and 97.7% versus 93.2%, respectively). In conclusion, UOS students demonstrated adequate knowledge, and possessed good attitudes and low-risk practices toward prevention of COVID-19. It is recommended that universities including UOS continue to use digital university communication platforms to regularly disseminate vital information in such emergencies.

## INTRODUCTION

Currently, the unprecedented coronavirus pandemic is hitting the whole world, including the United Arab Emirates (UAE). Coronaviruses are a large family of viruses that cause illnesses ranging from common cold to more severe diseases.^1^ There are several strains of this family, known to infect animals from different species. Previously, only two of them were found to infect humans, SARS-CoV^2^ and Middle East respiratory syndrome.^3^ However, in December 2019, there was an outbreak of a new disease in Wuhan city, China. It was later identified that the highly contagious disease, called COVID-19, was caused by a novel member of the family named SARS-COV-2.^4^ The virus soon spread to more than 200 countries and was declared as a global pandemic in March 2020 by the WHO.^5^

To date, there have been 29.5 million confirmed cases of COVID-19, including 932 thousands deaths reported worldwide.^6^ In the UAE, the first case was reported on January 29, 2020,^7^ and there are 82,000 cases diagnosed with 402 deaths and 71,500 recoveries till September 16, 2020.^8^

Substantial efforts have been made by researchers all over the world to develop a drug or vaccine that can cure this disease, but, unfortunately, there has not been any success yet, and infected patients continue to receive symptomatic treatment only.

The symptoms of the disease range from mild (such as fever, cough, and shortness of breath) to severe (such as pneumonia, SARS, and kidney failure).^9^ The battle against COVID-19 is still ongoing, and almost all countries implemented preventive measures such as strict infection control and partial/complete lockdown to curb the virus and “flatten the curve.” In the UAE, the lockdown took effect from the 22nd of March with partial movement restrictions for elderly and children younger than 12 years at the time of conducting the study.^10^

Public awareness and adherence to the recommendations play a major role in managing a crisis of this magnitude. Ways of preventing, managing, and minimizing the spread of COVID-19 have been discussed around the world. Current guidelines stress the importance of cleaning/washing hands, keeping social distance of at least 1 m, avoiding crowded places, avoiding touching the mouth and nose, and practicing respiratory hygiene; those with cough and other medical difficulties have been asked to seek medical attention.^1,9^ In addition, the CDC recommends covering the mouth and nose while coughing and sneezing, followed by immediate disinfection.^11^ The UAE had also issued a set of guidelines and preventive measures to fight the spread of COVID-19.^9^

The University of Sharjah (UOS) is located in Sharjah, one of the seven Emirates. It is one of the largest universities in the UAE, with a population of 15,000 students, 14 colleges, and more than 30 majors. There are mainly Arab and Emirati students who are resident in the UAE and/or Gulf Cooperation Council primarily. These students form a very important part of the young community and might influence the health and well-being of their families and friends. Furthermore, this population is the most socially active, both on social media and outside in the community, making them more vulnerable to contracting COVID-19 infection. During the time of the study, the population mainly visiting community spaces such as malls were young adults or the middle-aged working population because of the partial lockdown.^12^ Furthermore, the students from early March had all their classes shifted to the online mode, which reflected a new learning experience for the students.^13^ Also, information regarding COVID-19 was disseminated via online channels, including university website, LinkedIn, and Facebook pages.^14–16^ Because of the fact that these students have constant access to this information, it is important to evaluate if they are paying attention and adopting the information shared with them. Hence, assessment of their knowledge, attitudes, and practices (KAP) is crucial. Therefore, the aim of this study was to assess the KAP of the university students, as they represent a special part of the community with more autonomy but insufficient life experience which may impact their risk and that of others of COVID-19. Furthermore, comparing students from health-related (HR) and non–health-related (NHR) majors may be interesting to highlight similarities and/or differences in these population groups.

## MATERIALS AND METHODS

This cross-sectional survey was conducted during the partial lockdown in the UAE in the first 2 weeks of May 2020. A non-probability convenience sample included current students from the 14 colleges of the UOS who were invited to participate in the study. Because there was no previously published prevalence data related to COVID-19 in the UAE, sample size calculations were based on the assumption that the probability of having good knowledge, positive attitudes, and good practices toward preventing disease caused by coronavirus was 50%,^17^ at CI of 95%, with a precision of 5%; using this, the calculated sample size (*N*) was 384. To have a good representative sample from the different majors at the UOS and account for incomplete surveys, the questionnaire was sent by email to 1,300 students. By the end of the 2-week time limit to respond to the survey, 1,012 students responded and were included in the study. This sample size is 3-fold larger than that required (384 students).

A bilingual (Arabic and English) web-based questionnaire was sent to the students via a bulk email from the registration department as well as through a link on university digital platforms. The students were informed about the nature of the study and had the right to refuse participating in the study without any consequences. Those who were interested in participation had to grant permission for the data generated from their entries, and only then, they can proceed to fill out the questionnaire. The study protocol was approved by the Research and Ethics Committee at the UOS (REC-20-05-02-01).

The questionnaire was prepared based on the available information on the websites of the European and American Centers for Disease Control (ECDC and CDC, respectively) and the WHO. The questionnaire was reviewed by five academic experts in the field and revised based on their comments. The questionnaire was pretested on 40 students selected randomly from different colleges, and none of the respondents had comments that needed to be considered. The questionnaire included multiple-choice questions intended to assess the participants’ 1) sociodemographic characteristics, 2) knowledge, 3) attitudes, and 4) practices.^18,19^

The knowledge section included questions about the cause of the COVID-19, mode of transmission, common symptoms, complications, and the available treatment of the disease. The “attitudes” part included questions related to the seriousness of the disease and whether the student worries about suffering from or is at risk of COVID-19 infection, hence, disturbing the daily life. Moreover, some of the included questions explored the interest of students in knowing the methods of prevention, if the available information about COVID-19 was adequate and if the adoption of isolation measures and awareness could prevent or reduce the infection. The questions in the “practices” part were related to covering of the mouth and nose while sneezing, disposing of used tissues in the bin, and handwashing practices. Furthermore, this section inquired about the use of disinfectant and face mask, social distancing, contact with a person with COVID-19 and those with flu symptoms, touching or shaking hands, and seeking medical advice in case of any COVID-19 symptoms. Each item in the KAP sections marked correctly by the students based on the information provided by the Ministry of Health and Prevention (MOHAP)^9^ was given one point. The total scores of these items were then converted to percentages (0–100%). The scores of ≤ 60% were classified as poor knowledge, negative attitude, or high-risk practices; the scores of 60.1–80% were moderate knowledge, moderate attitude, or moderate-risk practices; and the scores of ≥ 80.1% were good knowledge, positive attitude, or low-risk practices.^18,19^

The collected data were analyzed using Statistical Package for the Social Sciences software, version 25.0 (SPSS, Chicago, IL). Categorical variables were expressed as frequencies and percentages, and continuous variables were expressed as mean ± SD. Independent *t*-test was used to compare the means of the knowledge scores of HR and NHR students. Statistical significance levels were set at *P* < 0.05. Chi-square test was used to explore the association between categorical variables. Sequential regression analysis was also carried out to explore the best predictors for the knowledge score.

**Figure 1.**
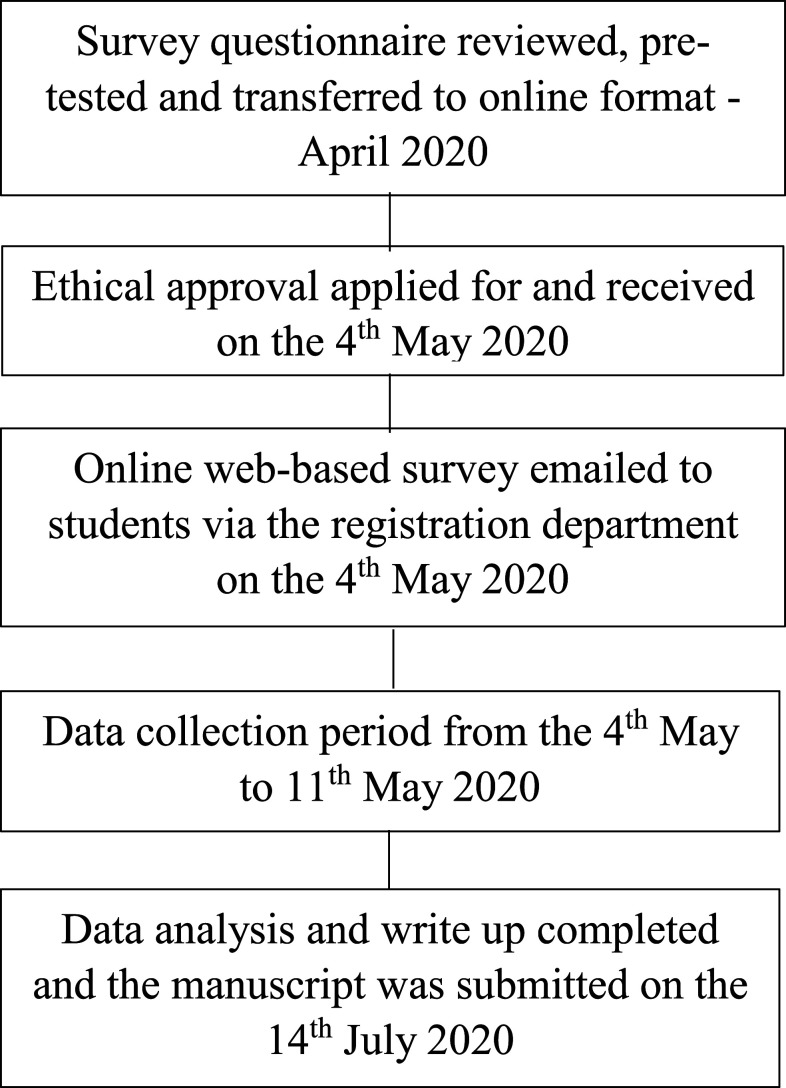
Time line of the study.

Figure 1 shows the time line of the study from the time of the revision to online transformation, to ethical application, and to sending the email to the participants until data analysis, write-up, and submission of the manuscript.

## RESULTS

The sample consists of 1,300 students from the UOS who were invited to participate in the study, and 1,012 students (19.4% males; 80.6% females) completed the questionnaire (response rate 77.8%).

As shown in Table 1, more than half (58%) of the students’ age ranged between 20 and 25 years. The majority of participants (88.9%) were Arab students, and 93.9% were single. About 51% and 41% of the students were living in villa and apartment, respectively. The distribution of students in HR (College of Health Sciences, Medicine, Dentistry, and Pharmacy) and NHR (all other colleges) majors was 47.5% and 52.5%, respectively.

**Table 1:** Sociodemographic characteristics of the participants (*N* = 1,012)

Variable		% (*N*)
Age-groups (years)		
	18–19.9	37.5 (380)
	20–25	58.0 (587)
	> 25	4.4 (45)
Gender		
	Males	19.4 (196)
	Females	80.6 (816)
Nationality		
	Arab	88.9 (900)
	Non-Arab	11.1 (112)
Marital status		
	Single	93.9 (950)
	Married	6.1 (62)
Place of residency		
	Villa	50.8 (514)
	Apartment	40.9 (414)
	University dormitories	8.3 (84)
Majors		
	Health related	47.5 (481)
	Non-health related	52.5 (531)

Table 2 shows the knowledge of the students toward COVID-19. Almost all the students (99.3%) knew about COVID-19, and their top three sources of information were Internet (any website unrelated to social messaging networks), social media (Facebook, Instagram, WhatsApp, and Snapchat) (85.2%) and mass media (56.5%), and official websites (WHO, CDC, MOHAP and other authorities in the UAE, UOS) (51%). A considerable percentage of students (46.5%) were not sure that the type of genetic materials in COVID-19 is RNA. However, 76.6% of the students were aware that COVID-19 infection is caused by a new member of the coronavirus. The majority of students (95.9%) were aware of cases of COVID-19 in the UAE, and 91.6% indicated that the history of travel was important to identify the risk of transmission of COVID-19. Only 10.4% of students indicated that there is no need for intensive and emergency treatment for a person with COVID-19. A large percentage of the students (91.8%) linked severe COVID-19 to death because of respiratory failure, with HR students more likely to report mortality of less than 5.0% than NHR students (40.1% versus 30.1%, *P* < 0.001). Almost all the students (98.1%) agreed that a person with COVID-19 should be immediately isolated, and more than three-fourths of the students (79.5%) concurred that complete recovery is possible in a person with COVID-19. Interestingly, 80.8% of the students agreed that the only effective treatment for COVID-19 is symptomatic.

**Table 2:** Knowledge of the participants toward COVID-19

			All (*N* = 1,012), % (*N*)	Health-related majors (*N* = 481), % (*N*)	Non–health-related majors (*N* = 531), % (N)	*P*-value
Do you know what COVID-19 is?						
		Yes	99.3 (1,005)*	99.4 (478)	99.2 (527)	0.8
		No	0.7 (7)	0.6 (3)	0.8 (4)	
What are your source(s) of COVID-19 information?						
	Internet and social media		85.2 (862)	84.4 (406)	85.9 (456)	0.51
	Mass media		56.5 (572)	54.7 (263)	58.2 (309)	0.26
	Official websites and scientific articles		51.0 (516)	63.0 (303)	40.1 (213)	< 0.001
	Friends and families		47.5 (481)	48.9 (235)	46.3 (246)	0.42
What is the cause of COVID-19 disease?						
	Virus		95.6 (967)*	99.6 (479)	91.9 (488)	< 0.001
	Bacteria		1.1 (11)	0.0 (0)	2.1 (11)	
	Not sure		3.4 (34)	0.4 (2)	6.0 (32)	
What is the type of genetic material in COVID-19?						
	DNA		20.0 (202)	24.5 (118)	15.8 (84)	< 0.001
	RNA		33.5 (339)*	45.5 (219)	22.6 (120)	
	Not sure		46.5 (471)	29.9 (144)	61.6 (327)	
Is COVID-19 caused by a new member of coronavirus?						
	Yes		76.6 (775)*	79.6 (383)	73.8 (392)	0.004
	No		15.7 (159)	15.6 (75)	15.8 (84)	
	Not sure		7.7 (78)	4.8 (23)	10.4 (55)	
Are there COVID-19 cases in the UAE?						
	Yes		95.9 (971)*	97.7 (470)	94.4 (501)	0.025
	No		1.0 (10)	0.6 (3)	1.3 (7)	
	Not sure		3.1 (31)	1.7 (8)	4.3 (23)	
Should the history of travel to areas experiencing transmission of COVID-19 be considered to identify persons at risk of having COVID-19?						
	Yes		91.6 (927)*	96.7 (465)	87.0 (462)	< 0.001
	No		2.2 (22)	0.8 (4)	3.4 (18)	
	Not sure		6.2 (63)	2.5 (12)	9.6 (51)	
Which of the following includes the mode(s) of COVID-19 transmission?						
	Coughing and sneezing		94.7 (958)*	95.6 (460)	93.8 (498)	0.19
	Kissing and shaking hands		90.9 (920)*	90.6 (436)	91.1 (484)	0.78
	Touching contaminated surfaces		88.8 (899)*	88.8 (427)	88.9 (472)	0.95
	Touching the nose or mouth		87.6 (887)*	90.2 (434)	85.3 (453)	0.02
	Saliva and nasal drip from the sick person with COVID-19		80.9 (819)*	87.3 (420)	75.1 (399)	< 0.001
	The use of objects owned by a COVID-19–infected person		80.2 (812)*	85.2 (410)	75.7 (402)	< 0.001
	Air		36.1 (366)	37.2 (179)	35.2 (187)	0.50
	Sexual route		32.2 (326)	28.3 (136)	35.8 (190)	0.01
	Consuming foods		17.6 (178)*	16.6 (80)	18.5 (98)	0.44
What is the incubation period for COVID-19?						
	≤ 14 days		60.5 (612)*	69.0 (332)	52.7 (280)	< 0.001
	> 14 days		34.6 (350)	29.1 (140)	39.6 (210)	
	Not sure		4.9 (50)	1.9 (9)	7.7 (41)	
Which of the following include people at high-risk group?						
	Elderly		93.9 (950)*	96.3 (463)	91.7 (487)	0.003
	Old people with comorbidity such as diabetes, cancer, and other chronic diseases		88.7 (898)*	92.9 (447)	84.9 (451)	< 0.001
	Children younger than 5 years		50.0 (506)	49.3 (237)	50.7 (269)	0.66
	Pregnant women		42.8 (433)*	47.4 (228)	38.6 (205)	0.005
	Adults		19.8 (200)	16.8 (81)	22.4 (119)	0.03
What are the most common symptoms of the disease?						
	Fever		94.4 (955)*	95.2 (458)	93.6 (497)	0.26
	Difficulty of breathing		91.7 (928)	94.2 (453)	89.5 (475)	0.007
	Dry cough		90.0 (911)*	91.9 (442)	88.3 (469)	0.06
	Sore throat		75.3 (762)	75.5 (363)	75.1 (399)	0.90
	Headache		71.1 (720)	71.3 (343)	71.0 (377)	0.91
	Tiredness		42.9 (434)*	45.3 (218)	40.7 (216)	0.14
	Runny nose		39.1 (396)	39.3 (189)	39.0 (207)	0.92
	Diarrhea		23.5 (238)	23.1 (111)	23.9 (127)	0.75
	Vomiting		16.0 (162)	15.8 (76)	16.2 (86)	0.86
	Blurred vision		8.8 (89)	6.4 (31)	10.9 (58)	0.01
	Skin rash		7.3 (74)	7.5 (36)	7.2 (38)	0.84
Should intensive and emergency treatment be given to diagnosed patients?						
	Yes		84.7 (857)	79.6 (383)	89.3 (474)	< 0.001
	No		10.4 (105)*	13.7 (66)	7.3 (39)	
	Not sure		4.9 (50)	6.7 (32)	3.4 (18)	
Which of the following includes complications of COVID-19 infection?						
	Bronchitis		51.5 (521)	51.7 (249)	51.2 (272)	0.86
	Sepsis		46.0 (466)*	43.0 (207)	48.8 (259)	0.07
	Pneumonia		41.6 (421)*	50.7 (244)	33.3 (177)	< 0.001
	Multi-organ failure		23.3 (236)*	26.8 (129)	20.2 (107)	0.01
	Hyperglycemia (high blood sugar)		4.3 (44)	6.7 (32)	2.3 (12)	0.001
If severe COVID-19 infection leads to death, is it usually caused by respiratory failure?						
	Yes		91.8 (929)*	95.2 (458)	88.7 (471)	< 0.001
	No		1.7 (17)	2.3 (11)	1.1 (6)	
	Not sure		6.5 (66)	2.5 (12)	10.2 (54)	
What is the approximate mortality rate in average patient infected with COVID-19? (%)						
	< 5		34.9 (353)*	40.1 (193)	30.1 (160)	< 0.001
	5–15		33.8 (342)	34.1 (164)	33.5 (178)	
	15–30		17.4 (176)	16.0 (77)	18.6 (99)	
	> 30		13.9 (141)	9.8 (47)	17.7 (94)	
Should COVID-19 cases be immediately isolated?						
	Yes		98.1 (993)*	99.6 (479)	96.8 (514)	0.004
	No		0.4 (4)	0.0 (0)	0.8 (4)	
	Not sure		1.5 (15)	0.4 (2)	2.4 (13)	
Could COVID-19 affect humans more than once in their lives?						
	Yes		57.8 (585)*	59.3 (285)	56.5 (300)	0.17
	No		17.8 (180)	18.9 (91)	16.8 (89)	
	Not sure		24.4 (247)	21.8 (105)	26.7 (142)	
Are antibiotics an effective medication in the treatment of COVID-19?						
	Yes		13.4 (136)	10.2 (49)	16.4 (87)	< 0.001
	No		60.0 (607)*	72.8 (350)	48.4 (257)	
	Not sure		26.6 (269)	17.0 (82)	35.2 (187)	
Is the current effective treatment for COVID-19 only by reducing symptoms?						
	Yes		80.8 (818)*	89.0 (428)	73.4 (390)	< 0.001
	No		8.1 (82)	6.0 (29)	10.0 (53)	
	Not sure		11.1 (112)	5.0 (24)	16.6 (88)	
Could COVID-19–infected people recover completely?						
	Yes		79.5 (805)*	78.4 (377)	80.6 (428)	0.06
	No		10.3 (104)	12.5 (60)	8.3 (44)	
	Not sure		10.2 (103)	9.1 (44)	11.1 (59)	
Is there a vaccine for COVID-19?						
	Yes		10.4 (105)	7.7 (37)	12.8 (68)	< 0.001
	No		66.9 (677)*	77.5 (373)	57.3 (304)	
	Not sure		22.7 (230)	14.8 (71)	29.9 (159)	
Knowledge score† (total is 31; 0 = 0% and 31 = 100%)			72.3 ± 10.62	76 ± 8.45	69 ± 11.31	< 0.001

* Good knowledge score.

† Using independent t-test to compare the knowledge score of the health-related and non–health-related students. As multiple responses were allowed, the total number of responses is greater than the number of surveyed participants, and percent of cases is displayed.

More HR students identified the cause of COVID-19 was a virus than NHR students (99.6% versus 91.6%, *P* < 0.001), with HR students being aware that it was caused by a new member of coronavirus (79.6% versus 73.8%, *P* = 0.004). A larger number of HR students identified travel history as being an important risk factor (HR versus NHR: 96.7% versus 87%, *P* < 0.001). Approximately 69% of HR students indicated that the incubation period for COVID-19 is ≤ 14 days versus 52.7% of NHR students (*P* < 0.001). Health-related students also reported that people with COVID-19 must be immediately isolated (HR versus NHR: 99.6% versus 96.8%, *P* = 0.004), and antibiotics are an ineffective treatment as compared with NHR students. A larger percentage of HR students indicated that the only effective treatment for COVID-19 was symptomatic (HR versus NHR: 89% versus 73.4%, *P* < 0.001).

Calculation of the knowledge score revealed that the mean score in percentage was 72.3 ± 10.62. Health-related students had significantly higher scores than NHR students (76% versus 69%, *P* < 0.001).

Table 3 illustrates the attitude of the UOS students toward COVID-19. About 85% of the students believe that COVID-19 is dangerous (HR versus NHR: students: 82.5% versus 87.6%, *P* = 0.024), and three-fourths of the students (76%) were worried about suffering from this infection. A large number of the students (74.1%) felt they are not at risk for COVID-19, and close to 90% stated that the disease had disturbed their daily lives. The majority (89.3%) were interested in obtaining more information about the methods of prevention of COVID-19, whereas only 61.6% considered that the available information is sufficient. Most students (91.4%) thought that adopting isolation measures could prevent COVID-19, and 95.7% believed that awareness could reduce COVID-19 infection.

**Table 3:** Attitude of the participants toward COVID-19

		All (*N* = 1,012), % (*N*)	Health-related majors (*N* = 481), % (*N*)	Non–health-related majors (*N* = 531), % (*N*)	*P*-value
Do you think that COVID-19 is a dangerous disease?					
	Yes	85.2 (862)*	82.5 (397)	87.6 (465)	0.024
	No	14.8 (150)	17.5 (84)	12.5 (66)	
Do you worry about suffering from COVID-19?					
	Yes	76.2 (771)	74.2 (357)	78.0 (414)	0.16
	No	23.8 (241)	25.8 (124)	22.0 (117)	
Are you at risk of getting COVID-19 infection?					
	Yes	25.9 (262)*	25.2 (121)	26.6 (141)	0.61
	No	74.1 (750)	74.8 (360)	73.4 (390)	
Has your daily life been disturbed by COVID-19?					
	Yes	89.2 (903)	88.8 (427)	89.6 (476)	0.65
	No	10.8 (109)	11.2 (54)	10.4 (55)	
Are you interested in knowing the methods of prevention of COVID-19?					
	Yes	89.3 (904)*	90.2 (434)	88.5 (470)	0.37
	No	10.7 (108)	9.8 (47)	11.5 (61)	
How would you describe the available information about COVID-19?					
	Sufficient	61.6 (623)	61.1 (294)	62.0 (329)	0.78
	Insufficient	38.4 (389)*	38.9 (187)	38.0 (202)	
Could the novel COVID-19 disease be prevented by adopting the isolation measures?					
	Yes	91.4 (925)*	91.1 (438)	91.7 (487)	0.71
	No	8.6 (87)	8.9 (43)	8.3 (44)	
Could the COVID-19 be reduced by awareness?					
	Yes	95.7 (968)*	96.9 (466)	94.5 (502)	0.07
	No	4.3 (44)	3.1 (15)	5.5 (29)	
Attitudes score (total is 6; 0 = 0% and 6 = 100%)		72 ± 14.5	71.72 ± 13.85	72.19 ± 15.11	0.29

* Positive attitudes.

Calculation of the attitude score revealed that the mean score was 72% ± 14.5%, with no significant differences between HR and NHR majors.

Table 4 demonstrates the practices of UOS students toward prevention of COVID-19. Most students (95.5%) cover their nose and mouth with elbow or tissue when coughing or sneezing, and 96.2% throw away the used tissue into the bin. The students wash their hands regularly when they come back home (92.3%), after toilet (90%), before eating (87.1%), after touching doorknobs or elevator buttons (85.5%), after touching personal items of someone who has a cough and/or cold (84%), after shaking hands with others (75.5%), and before touching eyes or nose (62%). Almost all the students (99.4%) used soap during handwashing, and about three-quarters wash hands before sleeping, with 20.7% washing their hands for greater than 20 seconds (HR versus NHR: 21.2% versus 20.2%, *P* = 0.015). The majority (86.2%) used disinfectant, disposable wipes, or hand gel for sanitization. About 90% of the students used face masks (HR versus NHR: 88.4% versus 92.3%, *P* = 0.034), with 71.3% using their mask in crowded areas, 46.8% always wearing masks, and 41.6% using masks only during fever, cough, or runny nose. The majority of the students (98.3%) avoid going to crowded places (HR versus NHR: 99.4% versus 97.4%, *P* = 0.013), and 95.4% practice social distancing (HR versus NHR: 97.7% versus 93.2%, *P* = 0.001). Almost all the students (99.5%) avoided contact with a person with COVID-19, with 94.2% avoiding touching or shaking hands and 90.4% avoiding contact with other people if they have flu-like symptoms. Nearly all (96%) the students concurred that they will seek medical advice if they experience any symptoms of COVID-19 infection.

**Table 4:** Practices of the participants toward COVID-19

		All (*N* = 1,012), % (*N*)	Health-related majors (*N* = 481), % (*N*)	Non–health-related majors (*N* = 531), % (*N*)	*P*-value
Do you cover your nose and mouth with elbow or tissue when coughing and sneezing?					
	Yes	95.5 (966)*	96.7 (465)	94.4 (501)	0.07
	No	4.5 (46)	3.3 (16)	5.6 (30)	
Do you throw away the used tissue into the bin?					
	Yes	96.2 (974)*	97.1 (467)	95.5 (507)	0.18
	No	3.8 (38)	2.9 (14)	4.5 (24)	
When do you wash your hands?					
	When you come back home	92.3 (934)*	92.9 (447)	91.7 (487)	0.47
	After toilet	90.0 (911)*	94.6 (455)	85.9 (456)	< 0.001
	After touching doorknobs or elevator buttons	85.5 (865)*	85.2 (410)	85.7 (455)	0.84
	Before eating	87.1 (881)*	89.4 (430)	84.9 (451)	0.03
	After touching the personal items of someone who has a cough and/or cold	84.0 (850)*	83.6 (402)	84.4 (448)	0.73
	After shaking hands with others	75.5 (764)*	73.4 (353)	77.4 (411)	0.14
	Before touching eyes or nose	62.0 (627)*	61.5 (296)	62.3 (331)	0.79
Do you use soap in hand washing?					
	Yes	99.4 (1,006)*	99.2 (477)	99.6 (529)	0.35
	No	0.6 (6)	0.8 (4)	0.4 (2)	
Do you wash your hands before sleeping?					
	Yes	74.2 (751)	72.8 (350)	75.5 (401)	0.32
	No	25.8 (261)*	27.2 (131)	24.5 (130)	
How much time do you spend washing your hands? (seconds)					
	Less than 10	15.5 (157)	12.1 (58)	18.6 (99)	0.015
	10–20	63.8 (646)	66.7 (321)	61.2 (325)	
	More than 20	20.7 (209)*	21.2 (102)	20.2 (107)	
Do you use disinfectant, disposable wipes or hand gel to wash your hands?					
	Yes	86.2 (872)*	86.1 (414)	86.3 (458)	0.93
	No	13.8 (140)	13.9 (67)	13.7 (73)	
Do you use a face mask?					
	Yes	90.4 (915)*	88.4 (425)	92.3 (490)	0.034
	No	9.6 (97)	11.6 (56)	7.7 (41)	
When do you use a face mask?					
	At crowded areas	71.3 (722)	74.2 (357)	68.7 (365)	0.05
	Always wearing face mask	46.8 (474)	39.7 (191)	53.3 (283)	< 0.001
	When having fever, cough, or runny nose	41.6 (421)	45.3 (218)	38.2 (203)	0.02
Do you avoid going to crowded places?					
	Yes	98.3 (995)*	99.4 (478)	97.4 (517)	0.013
	No	1.7 (17)	0.6 (3)	2.6 (14)	
Do you practice social distancing?					
	Yes	95.4 (965)*	97.7 (470)	93.2 (495)	0.001
	No	4.6 (47)	2.3 (11)	6.8 (36)	
Do you avoid contact with COVID-19–infected case?					
	Yes	99.5 (1,007)*	99.8 (480)	99.2 (527)	0.21
	No	0.5 (5)	0.2 (1)	0.8 (4)	
Do you avoid touching or shaking hands?					
	Yes	94.2 (953)*	95.2 (458)	93.2 (495)	0.17
	No	5.8 (59)	4.8 (23)	6.8 (36)	
If you have flu symptoms, do you avoid contact with other people?					
	Yes	90.4 (915)*	91.5 (440)	89.5 (475)	0.27
	No	9.6 (97)	8.5 (41)	10.5 (56)	
Would you seek medical advice if you experience any symptoms of COVID-19 disease?					
	Yes	96.0 (972)*	95.4 (459)	96.6 (513)	0.33
	No	4.0 (40)	4.6 (22)	3.4 (18)	
Practices score (total is 20; 0 = 0% and 20 = 100%)		85.6 ± 11.8	86.04 ± 11.37	85.25 ± 12.21	0.62

* Low-risk practice.

Calculation of the practice revealed that the mean score was 85.6% ± 11.8%, with no significant difference in score between HR and NHR students.

Table 5 depicts the sequential regression analysis using the knowledge score as a dependent variable and gender, nationality, or study majors as independent variables. It shows that model 3 has the largest and significant *R* square change (0.108, *P* < 0.001), and the two significantly associated variables were the gender and study majors (*B* = −0.51, *P* = 0.04, 95% CI: −1.01 to −0.01 and *B* = −2.23, *P* < 0.001. 95% CI: −2.62 to −1.83, respectively). The results show that males and HR majors had higher knowledge score; however, study major was the best predictor for the knowledge score.

**Table 5:** Sequential regression analysis using knowledge score as a dependent variable

	Model 1				Model 2				Model 3			
	(*R*^2^ = 0.000, R^2^ change = 0.000)				(*R*^2^ = 0.001, *R*^2^ change = 0.001)				(*R*^2^ = 0.109, *R*^2^ change = 0.108)*			
	*B*	*P*-value	95% CI		*B*	*P*-value	95% CI		*B*	*P*-value	95% CI	
			Lower	Upper			Lower	Upper			Lower	Upper
Gender	0.17	0.52	−0.34	0.68	0.168	0.52	−0.034	0.68	−0.51	0.04	−1.01	−0.01
Nationality	–	–	–	–	0.30	0.36	−0.35	0.95	0.13	0.67	−0.48	0.74
Majors	–	–	–	–	–	–	–	–	−2.23	< 0.001	−2.62	−1.83

* *P* < 0.001.

## DISCUSSION

Following the COVID-19 pandemic, as part of the Ministry of Education and MOHAP guidelines for all universities and students in the UAE, all teaching (including theory, laboratory, and clinical courses) were changed from 100% face-to-face to 100% online delivery. Furthermore, guidelines were provided to all university students, faculty, and staff in mid-March, for enhancing knowledge and practices to prevent COVID-19 infection in the community. Students form a very important and influential population group both for their peers and families. Hence, their perspective is crucial to evaluate current KAP toward COVID-19 to provide recommendations for improvements in these areas. This study was conducted to evaluate and compare the KAP of HR and NHR UOS students toward COVID-19. Of the 1,012 students who completed the KAP questionnaire, most were female Arab students. Student responses from HR and NHR majors were almost equal. Although the students from all majors had acceptable knowledge, positive attitudes, and adequate practices, there was a scope for improvement in certain key areas that will be highlighted in the following text.

Regarding the knowledge of COVID-19, more than 80% of students relied on the Internet and social media as the main source of information as opposed to more traditional sources such as newspapers, television channels, radio, or scientific websites. This could be attributed to the fact that the student population is young and have their mobile devices with Internet availability almost all the time.^20^ Although the UOS sent announcements via email, on their Facebook, Instagram, and Linked-in pages^14–16^ for the university, the number of students accessing official websites is limited. Most students who relied on social media may have become victims of misinformation and fabricated knowledge from unreliable sources. The university needs to concentrate efforts to direct students to credible social media platform pages. Most of the students attributed “a virus” as the cause of COVID-19 disease. However, almost two-thirds of students from all majors were not able to identify RNA as the type of genetic material of the virus. Similar findings were reported by another study conducted in the Jordanian students.^18^ More HR students were able to identify the genetic material type with HR students more likely to report mortality of less than 5% than NHR students (40.1% versus 30.1%, *P* < 0.001) possibly because their curriculum includes information regarding viruses, or they are more interested in learning health-related information.

Most of the students indicated that history of travel was important to identify the risk of COVID-19 transmission; however, more HR students indicated this. A significant number of the students reported good awareness about the modes of transmission, although a little over 50% were aware of the incubation period for COVID-19. This is very similar to that found in Pakistani University community.^21^ Recent evidence indicates that aerosols related to certain medical procedures carried out on people with COVID-19 could be a potential source of infection if inhaled by those who are not wearing personal protective equipment. Furthermore, aerosol transmission linked to infection in closed spaces can also be a source of infection^22^; however, air was significantly identified as a mode of transmission in the current study. More awareness needs to be raised, and further research needs to be conducted in this area.

More than 80% of all the students were able to identify elderly and those with comorbidities as a high-risk group for COVID-19; however, only approximately 40% identified that pregnant women are at high risk. Half of the students identified children younger than 5 years to be at high risk, which is contrary to that reported by the CDC and WHO.^9,11,18,23^ This could be attributed to the movement restrictions applied to children for malls and public places.^12^ It is recommended that public programs by the government focus on enhancing awareness of the high-risk groups for COVID-19.

Almost all the students were aware of common symptoms such as fever and dry cough as identified by the MOHAP^9^; however, less than half of the students were aware of tiredness as being an additional common symptom for COVID-19. It has been extensively reported that more than 80% of people with COVID-19 have mild symptoms and recover without the need for intensive care.^23^ Nevertheless, almost 85% of the students reported a need for intensive and emergency treatment for all persons with COVID-19, which is another area that students need further awareness about.

Complications of COVID-19 focus on acute respiratory failure, pneumonia, acute respiratory distress syndrome, acute liver injury, acute cardiac injury, secondary infection, acute kidney injury, septic shock, and disseminated intravascular coagulation.^24^ About half the students were aware of the most common complications associated with COVID-19. This is lower than that reported by a study in Jordan with university students. In our study, 46% of the students reported sepsis as being a complication as opposed to a Jordanian study which reported only 5% of students identifying sepsis as a complication.^18^ Only one-fourth of our students identified that multi-organ failure is a complication of COVID-19. This might be attributed to the fact that multi-organ failure is seen only in very severe COVID-19 cases, which constitute only 4% of infected people.^23,25^

It is well established that respiratory-related complications can result in a high mortality rate among COVID-19–infected patients, and the bulk of students in our study were able to relate severe COVID-19 to death because of respiratory failure, similar to the findings from Jordan^18^

Almost all the students demonstrated knowledge regarding immediate isolation of a person with COVID-19; however, only a little over half knew that COVID-19 could affect humans more than once in their lifetime. This is an important area to raise awareness as it might result in students not following social distancing, or more appropriately referred to as physical distancing,^21^ and other preventive measures once they have recovered from COVID-19.

Three-fourths of HR students reported that antibiotics are ineffective in treating COVID-19 as opposed to less than half of NHR students. This highlights that the differences in student majors may greatly impact health-related knowledge, and that awareness should be focused on those not in the healthcare sector.

About four-fifths of the students knew that complete recovery is possible, and till the time of writing this article, there is no available vaccine for COVID-19 or established effective treatment, and the primary management is symptomatic.^26^ However, more HR students were aware of the above information as opposed to approximately half of the NHR students. This again highlights the importance of awareness to be focused on the NHR sector.

Our students demonstrated moderate knowledge (72%) comparable to medical students from Jordan,^27^ and greater than that reported by Saudi^28^ and Pakistani students.^21^ However, our students reported lower knowledge than other majors’ students from Jordan^18^ and Iranian public.^29^ Furthermore, sequential regression indicated that gender and study major are significantly associated with the knowledge score, with the latter being the best predictor as expected. Despite providing adequate information on the UOS website, media channels, and other sources, this study highlights the need to provide further knowledge awareness related to COVID-19 particularly in NHR majors.

Exploring the attitudes of the UOS students revealed that a large number believed that COVID-19 was dangerous, and about three-quarters were worried about suffering from the infection. This finding is very similar to the one in Pakistani students.^21^ Interestingly, the same percentage of students (three-quarters) believed that they were not at risk for getting COVID-19, which could be attributed to the study being conducted during the UAE lockdown and Ramadan (a periodic month of fasting every year for the Muslim community), both of which limited students’ outings and subsequent contact with others.

Most of them were interested in obtaining more information about prevention of COVID-19, and only more than 50% felt that they had sufficient information. This gap needs to be bridged so that attitudes and practices can be improved.

Regarding the attitude score (72%), our students demonstrated moderate positive attitudes toward COVID-19; however, these are lower than the percentage score among Jordanian students,^19^ suggesting the need to raise awareness in this important area.

Based on the CDC recommendations^30^ for using cloth masks in public places, almost all the students follow these practices when visiting public places. Furthermore, they also make sure to cover their nose and mouth while sneezing, and dispose of tissues appropriately. Regarding handwashing, students do wash it regularly after coming home, or using the washroom, but almost 60% do wash their hands regularly before touching the eyes or nose, which is a preventative recommendation.^30^ Furthermore, the recommendation to wash hands for more than 20 seconds^31^ is only adhered by one-fifth of the students. The majority use disinfectant, disposable wipes, or hand gel for sanitization. This highlights areas where practices may be improved.

Most of the students avoid going to crowded places and practice social distancing. All students avoid contact with a person with COVID-19, including avoiding touching or shaking hands and avoiding contact with other people if they have flu-like symptoms. All the students concurred that they would seek medical advice if they experience any symptoms of COVID-19 infection. The students demonstrated a practice score of 85% reflecting low-risk practices of the students to prevent spread or risk of COVID-19 infection. This is similar to the finding in Jordanian students^19^; however, this is different as compared with other studies reporting unsatisfactory practices.^21^ This may be due to the fact that the population is young students who are up to date with the information.

In conclusion, large-scale pandemics, as we are apparently experiencing, test the integrity of awareness campaigns conducted regularly, especially on online platforms in a socially active population like university students. In our study, all students demonstrated adequate knowledge as evident by their knowledge scores. Furthermore, it is fulfilling to discover that the students also possess good attitudes and low-risk practices toward prevention of COVID-19. However, it is apparent that HR students fared better in all categories as than NHR students, especially in the knowledge domain. Unnecessary emergency and intensive treatment for every person with COVID-19, identification of the high-risk groups, particularly pregnant women, and the possibility of recurrence of COVID-19 infection highlighted three areas of focus for awareness campaigns for the UOS students, particularly those in NHR majors. The findings of this study reflect the importance for institutions like universities to develop appropriate educational programs and provide protective health measures for their faculty, staff, and students, especially considering the possibility of physical reopening in the spring semester. These might possibly include posters, seminars, information via social channels on good respiratory etiquette, handwashing practices, and physical distancing to enhance safer lifestyles and prevent COVID-19 transmission.
